# White Matter Hyperintensities Are Associated With Severity of Essential Tremor in the Elderly

**DOI:** 10.3389/fneur.2021.694286

**Published:** 2021-06-28

**Authors:** Jos S. Becktepe, Johannes Busse, Ulf Jensen-Kondering, Inken Toedt, Stephan Wolff, Kirsten E. Zeuner, Daniela Berg, Oliver Granert, Günther Deuschl

**Affiliations:** ^1^Department of Neurology, University Hospital Schleswig-Holstein, Christian-Albrechts University, Kiel, Germany; ^2^Department of Neuroradiology, University Hospital Schleswig-Holstein, Christian-Albrechts University, Kiel, Germany

**Keywords:** white matter hyperintensities, essential tremor, senile tremors, accelerometric tremor frequency, tremor severity

## Abstract

**Background:** Essential tremor (ET) occurs with steeply increasing prevalence in the elderly, and apart from disease duration, age is independently associated with an increase of tremor amplitude and a decrease of frequency. White matter hyperintensities (WMHs) are a common finding in the elderly, and their role in the pathophysiology of ET is unknown. The aims of this study were to examine whether ET patients differ in their total or region-specific WMH volumes from healthy controls and to determine the impact of WMH on tremor characteristics.

**Methods:** A total of 47 elderly ET patients with a mean age of 72 years and 39 age-matched healthy controls underwent a thorough clinical assessment and 3T MRI. Total WMH volumes were derived from T2-weighted fluid-attenuated inversion recovery (FLAIR) MR images. Additionally, region of interest-based WMH volumes for the Johns Hopkins University (JHU) white matter tracts and labels were calculated, and WMHs were assessed semiquantitatively using the Fazekas scale.

**Results:** Essential tremor patients and healthy controls did not differ in their total or tract-specific WMH volumes or Fazekas scores. However, WMH volume was significantly positively correlated with tremor severity on the TETRAS scale, and there was a significant negative correlation with the mean accelerometric tremor frequency. In a multiple linear regression model including disease duration, age, and age-adjusted total WMH volume, only the WMH volume significantly predicted tremor severity, while age and disease duration were not significant.

**Conclusion:** We found evidence for a direct association between WMH volume and tremor severity. If confirmed by larger studies, our findings could explain the well-known relation between age and tremor severity.

## Introduction

Essential tremor (ET) is the most common movement disorder, with steeply increasing prevalence in the elderly (1). According to the current classification, ET is considered a syndrome with clinical features described in axis I and the etiology in axis II (2). Although the etiology for the syndrome (axis II) remains unclear in the majority of patients, pathological oscillations within the cerebello-thalamo-cortical circuit are proposed as a common pathophysiologic correlate (3). The source for these oscillations has not been determined yet, but several imaging and pathological studies point toward a central role of the cerebellum for the pathogenesis (4–7).

Using linear regression analyses, epidemiologic studies have shown that apart from disease duration, age is independently associated with an increase in tremor severity as measured with clinical tremor scores and a decrease in accelerometric tremor frequency (8–11). Furthermore, older age of tremor onset is associated with a more rapid tremor progression, and patients with a tremor onset after the age of 60 have a shorter life expectancy (12–15). Such late-onset patients also show electrophysiological parameters for earlier aging, such as a prolonged latency of the pupillary response (15) and a different cerebral network of tremor (16). The underlying mechanisms for this relationship between biological aging and tremor progression are not understood yet, but a subtype of aging-related (senile) tremor is one explanation.

White matter hyperintensities (WMHs) on T2-weighted MRI are a very common finding in the elderly (17). Pathologically, they correspond to areas of demyelination and gliosis, mainly resulting from chronic diffuse subclinical ischemia (18). White matter hyperintensities affect cognitive functioning in normal aging and also in patients with mild cognitive impairment and dementia (17). Furthermore, there is growing evidence that WMHs affect motor performance in the elderly in several ways: WMHs are associated with a higher risk of developing gait disturbances in healthy elderly persons (19). In Parkinson's disease patients, WMHs are associated with an increased risk of progression from mild parkinsonian signs to severe gait and balance impairment, bradykinesia, and rigidity (20), and they mediate the effect of autonomic dysfunction on future cognitive decline (21).

To the best of our knowledge, only one study has examined the occurrence of WMHs in ET patients, finding that ET was associated with greater total WMH volume and greater cerebellar WMH volume compared to those in healthy controls (22). But these results have never been confirmed within a sample of ET patients that was diagnosed according to the current International Parkinson and Movement Disorder Society (IPMDS) tremor classification.

The objectives of this study were (1) to examine whether patients with ET differ in their total or region-specific WMH volumes from healthy, age-matched controls and (2) to examine the impact of WMHs on certain tremor characteristics (tremor amplitude, frequency, tremor score).

## Participants and Methods

### Clinical Assessment

Between 2017 and 2019, a sample of 55 ET patients and 41 healthy controls with at least 60 years of age was consecutively recruited from our outpatient clinic. Of these, eight patients and two healthy controls had incidental MRI findings and were excluded from all further analyses (see below).

Inclusion criteria for patients were a diagnosis of ET or ET plus according to the current diagnostic criteria of the IPMDS (2) and a minimum age of 60 years. Exclusion criteria were a history of clinically evident stroke, dementia, or other neurological diseases apart from ET or incidental MRI findings apart from WMH (see imaging exclusion criteria). Healthy controls were either spouses of patients (*n* = 8) or individuals who were registered within an in-house database for voluntary participation in research studies (*n* = 33). Patients and controls underwent a complete neurological examination by a movement disorder specialist, and patients were asked to pause any antitremor medication for at least 24 h before the examination. The presence of vascular risk factors (arterial hypertension, hyperlipidemia, diabetes mellitus) and vascular disease defined as a history of coronary artery disease (myocardial infarction, angina, atrial fibrillation, congestive heart failure) or cerebrovascular disease (carotid endarterectomy, carotid stent) was assessed based on a thorough review of the participant's medical history and medications. For each patient, a vascular burden score was calculated from vascular risk factors and vascular diseases, ranging from 0 to 5 (i.e., the sum of hypertension, hyperlipidemia, diabetes mellitus, coronary artery, and cerebrovascular diseases) (23). Cognitive functioning was tested using the Montreal Cognitive Assessment (MoCA) (24). In addition, semantic and verbal fluency was assessed with the Regensburger Wortflüssigkeits-Test (RWT), 2-min testing per task, no counting of errors (25). In all patients, tremor severity was rated according to the Essential Tremor Rating Assessment Scale (TETRAS) and by polygraphic tremor analysis (26).

The study was approved by our local ethics committee, and all individuals gave written informed consent prior to participation.

### Electrophysiological Tremor Assessment

Tremor amplitude and frequency were assessed by polygraphic tremor analysis (27). Patients were seated in a comfortable chair in a slightly supine position. Both forearms were supported by firm armrests up to the wrist joints. During measurement, the hands were held outstretched against gravity. Tremor was recorded for 30 s by surface electromyography (EMG) from the extensor and flexor carpi ulnaris muscles using silver chloride electrodes and a piezoresistive accelerometer, which was placed on the third metacarpal bone about 2 cm proximal to the metacarpophalangeal joint bilaterally. All data were sampled at 800 Hz. The EMG was bandpass filtered between 50 and 350 Hz and full wave rectified. Spectral analysis was performed using a standard algorithm implemented in a commercially available tremor analysis software [as described in Lauk et al. (27)]. As a measure of tremor amplitude, the logarithmic total power of the accelerometrically measured tremor spectra was calculated. The greatest peak power was considered to reflect the tremor frequency.

### Magnetic Resonance Image Acquisition

Magnetic Resonance Image acquisitions were performed on a 3-Tesla whole-body MRI scanner (Achieva; Philips, Best, Netherlands) equipped with a 32-channel head coil. The imaging protocol consisted of the following:

I) A T1 MPRAGE sequence with a spatial resolution of 1.05 × 1.05 × 1.2 mm, 170 slices, a field of view of 256 × 256 mm, TR = 6.6 ms, TE = 3.1 ms, and a flip angle = 9°.
II) A T2 FLAIR sequence with a spatial resolution of 0.43 × 0.43 × 2.0 mm, 57 slices, a field of view of 528 × 528 mm, TR = 12,000 ms, TE = 160 ms, TI = 2,850 ms, and a flip angle of 90°.
III) A diffusion tensor imaging (DTI) dataset with 64 directions, a spatial resolution of 1.67 × 1.67 × 1.9 mm, a field of view of 144 × 144 mm, TR = 8,200 ms, TE = 75 ms, and a flip angle of 90°. Diffusion-weighted images were acquired in four consecutive scan sessions with intermitted B0 images. At the end of the acquisition, a reference scan with opposing polarities of the phase-encoding was added to correct susceptibility-induced distortions.

Additionally, a hemosensitive T2^*^-weighted sequence was acquired for clinical purposes.

### Imaging Exclusion Criteria

All FLAIR and T2^*^ sequences were screened by a board-certified radiologist with 10 years of experience in neuroradiology for incidental findings. Eight patients and two controls had incidental MRI findings and were excluded from further analyses: four patients had incidental embolic or lacunar stroke, one patient had a giant subarachnoid cyst preventing further processing of the MRI images, one patient had imaging evidence of idiopathic normal pressure hydrocephalus, two patients and two control subjects fulfilled imaging criteria for probable cerebral amyloid angiopathy.

### Semiquantitative White Matter Hyperintensity Assessment

Periventricular and deep white matter signal hyperintensities were assessed semiquantitatively by a blinded examiner using the Fazekas scale on FLAIR images (Table 1) (28).

**Table 1:** Baseline characteristics.

	**ET**		**Controls**		***t*-test**
	***n*** **=** **47**		***n*** **=** **39**		
	**Mean**	**Std. Deviation**	**Mean**	**Std. Deviation**	***p*-value**
Age	72.36	6.76	71.54	6.96	0.567
School years	10.37	1.92	10.87	1.60	0.194
Body mass index	25.54	3.52	26.94	5.55	0.173
MoCA	24.28	2.84	26.02	2.70	**0.005**
MoCA age adjusted	−0.15	0.83	0.38	0.86	**0.004**
RWT semantic	31.18	9.79	34.57	8.84	0.100
RWT phonematic	18.31	8.61	20.31	8.50	0.285
BDI	3.36	4.29	2.37	3.21	0.244
TETRAS part 1	22.71	7.05			N/A
TETRAS part 2	19.67	4.06			N/A
	**Median**	**Range**	**Median**	**Range**	**Mann–Whitney–U-test**, ***p*****-value**
Vascular burden score (0–5)	1	4	1	3	0.128
Fazekas periventricular white matter (0–3)	1	3	1	2	0.301
Fazekas deep white matter (0–3)	1	2	1	2	0.951

*BDI, Beck's Depression Inventory; MoCA, Montreal Cognitive Assessment; RWT, Regensburger Wortflüssigkeits-Test; TETRAS, Tremor Research Group Essential Tremor Rating Assessment Scale; Significant values are marked in bold*.

### Automated Total and Region of Interest-Based White Matter Hyperintensity Assessment

1. FreeSurfer segmentation: A volumetric segmentation was applied to the structural T1 image using the FreeSurfer image analysis pipeline (recon-all). The technical details of these procedures are described in prior publications (29).
2. FLAIR-based WMH detection: Image intensity correction (bias field correction) was applied to the T1 and FLAIR images to get uniform image intensities (30). A rigid transformation estimated in a FLAIR to T1 registration step was used to align and transfer the FreeSurfer segmentation to the FLAIR image matrix (31). Based on FreeSurfer tissue segmentations projected onto the voxel of the FLAIR image, mean and standard deviation of the FLAIR intensities of gray matter were calculated, and an intensity threshold for WMHs was automatically determined by choosing the first upper quantile of the gray matter FLAIR intensities as a threshold for WMH regions.
3. DTI preprocessing: Images from the four consecutive DTI sequences were merged, and a brain mask was calculated using the FSL bet2 software (32). The FSL topup was applied to the B0 images with opposing polarities to estimate a distortion map. The method is described in Andersson et al. (33) and implemented in FSL (34). The distortion map and eddy correction were then applied to the DTI data to correct spatial and eddy distortions. After this correction, a new brain mask was calculated that respects the corrected image geometry. The corrected DTI dataset and the new brain mask were then piped into FSL's DTIFIT to calculate individual fractional anisotropy (FA) and mean diffusivity (MD) maps.

**Figure 1:**
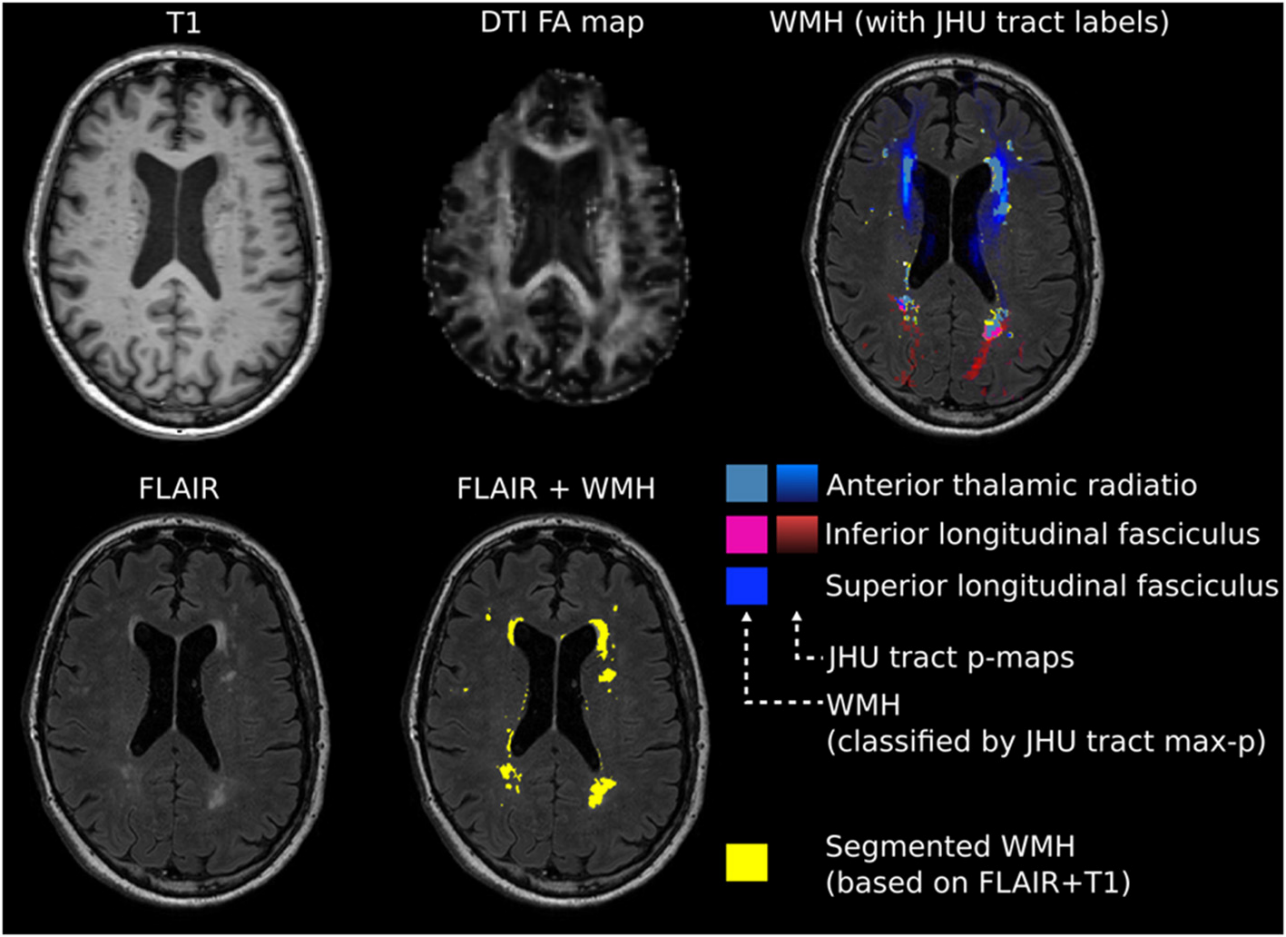
Exemplary image processing of a single patient for region of interest (ROI)-based white matter hyperintensity (WMH) assessment. Intensity threshold for WMHs was automatically determined by choosing the first upper quantile of the gray matter as a threshold. Individual fractional anisotropy (FA) maps were coregistered with the Johns Hopkins University (JHU) atlas FA maps. Whole-brain and tract-specific WMH volumes were automatically calculated in mm3.

The individual FA maps were registered with the JHU atlas FA maps to assign detected WMH voxels to specific JHU tracts using the JHU max probability map (JHU ICBM tracts maxprob thr0 1 mm) (35). Additionally, WMH voxels were assigned to the JHU labels atlas (36). Whole-brain and tract-/label-specific WMH volumes were automatically calculated in mm3. Figure 1 summarizes major steps of the preprocessing pipeline.

### Statistical Analyses

IBM SPSS Statistics (Version 24.0; IBM Corp., Armonk, NY) was used for statistical analyses. *t*-Tests were applied for group comparisons of baseline clinical characteristics and total or ROI-based WMH volumes. Nonparametric Mann–Whitney–U-test was applied for group comparisons of the Fazekas scale and vascular burden score. Pearson's chi-square-test was used to test categorical variables. Results were considered significant for *p* < 0.05.

To examine whether WMHs are associated with tremor severity and frequency in ET patients, a multiple linear regression model was compiled. The total WMH volume was adjusted for age to allow including both as independent variables into this model. To identify strategic white matter tracts in which WMHs are associated with tremor severity independently of global WMH volume, assumption-free ROI-based analyses were performed. Therefore, the tract-specific WMH volume was adjusted for total WMH volume, and bilateral JHU tracts/labels were merged into a single ROI. The regional WMH volumes of these 11 white matter tracts/27 JHU labels were entered separately into linear regression models as independent variables with TETRAS part 2 scores or tremor frequency as dependent variables. Bonferroni correction was applied for multiple comparisons.

## Results

### Baseline Characteristics

A sample of 47 ET patients and 39 healthy controls, both groups >60 years of age, was included in the study. Table 1 summarizes the baseline characteristics of the study cohort. In 20 of the 47 ET patients, additional neurological signs of uncertain significance were found (e.g., questionable dystonic postures, mild gait ataxia, etc.) and these patients were labeled as ET plus accordingly. Since no group differences were found between patients with and those without additional soft signs regarding total or ROI-specific WMH volumes, all ET patients were pooled for further analyses to increase statistical power.

Essential tremor patients had a significantly lower MoCA score than that in healthy controls even after correction for age (Table 1). Both groups did not differ in their verbal fluency measures. Patients and controls did not differ in their vascular burden score (*p* = 0.128, Mann–Whitney–U-test), although patients had a slightly wider range (Table 1).

Within the group of ET patients, the TETRAS motor score (TETRAS part 2) was significantly negatively correlated with tremor frequency (mean value of left and right upper extremity; *r* = −0.39, *p* = 0.009), but there was no significant correlation of TETRAS part 2 with the logarithmic accelerometric total power (mean value between left and right upper extremity; *r* = 0.166, *p* = 0.326). However, total power and tremor frequency were significantly negatively correlated (*r* = −0.33, *p* = 0.043).

### White Matter Hyperintensity Volumes

**Figure 2:**
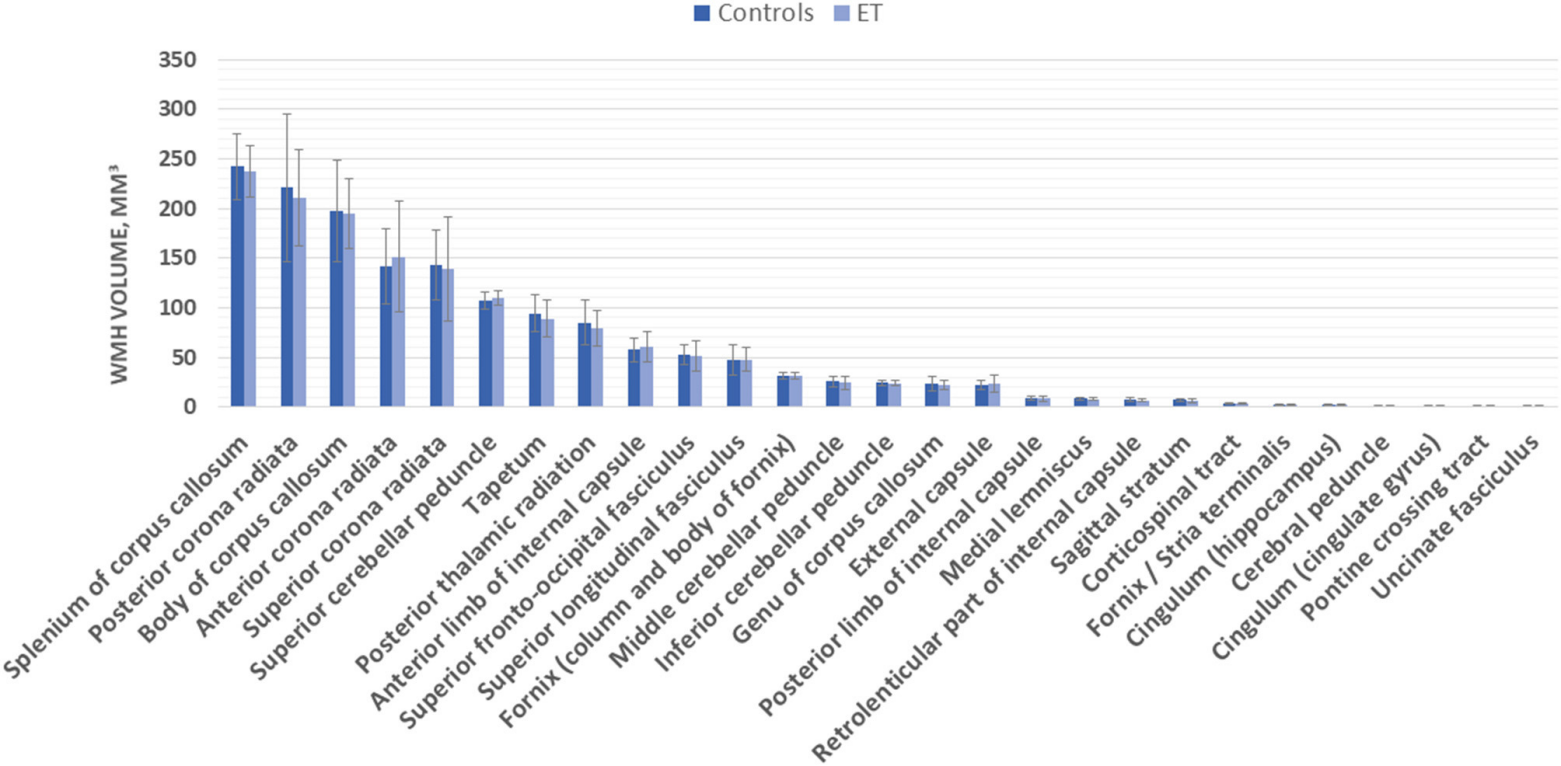
Region of interest (ROI)-specific white matter hyperintensity (WMH) volume (in mm3) in essential tremor (ET) patients and controls.

Vascular burden score was significantly correlated with total WMH volumes (Spearman rho = 0.270, *p* = 0.012). Patient groups and healthy controls did not differ significantly with regard to the total volume of WMH nor to the ROI-specific WMH volume (mean values of left/right side ROI; Figure 2). These results remained not significant when taking the vascular burden score, age, and age-adjusted MoCA score as covariates into the analysis. The semiquantitative assessment of the Fazekas scale confirmed these results (no significant group differences; Table 1). When splitting the sample of ET patients into a group with early (<40 years, *n* = 23) and late (≥60 years, *n* = 12) onset of symptoms, no significant differences for total or ROI-specific WMH volumes were found between these groups or in comparison to healthy controls after age correction. Both groups also did not differ regarding TETRAS part 2 scores or mean tremor frequency, although patients with late symptom onset had a significantly shorter disease duration (11.4 vs. 51.3 years, *p* < 0.001).

### White Matter Hyperintensities and Tremor Characteristics

Age and disease duration were not significantly correlated with each other (*r* = −0.267, *p* = 0.07), and both age and disease duration were not directly correlated with tremor severity on the TETRAS scale (correlation analysis for age/TETRAS: *r* = 0.08, *p* = 0.590; duration: *r* = −0.03, *p* = 0.858) nor the logarithmic total power of postural tremor (age: *r* = −0.146, *p* = 0.297; duration: *r* = 0.060, *p* = 0.667). However, age was significantly correlated with the total WMH volume (*r* = 0.328, *p* = 0.009). Therefore, the WMH values were controlled for age to allow including both into a linear regression model.

**Figure 3:**
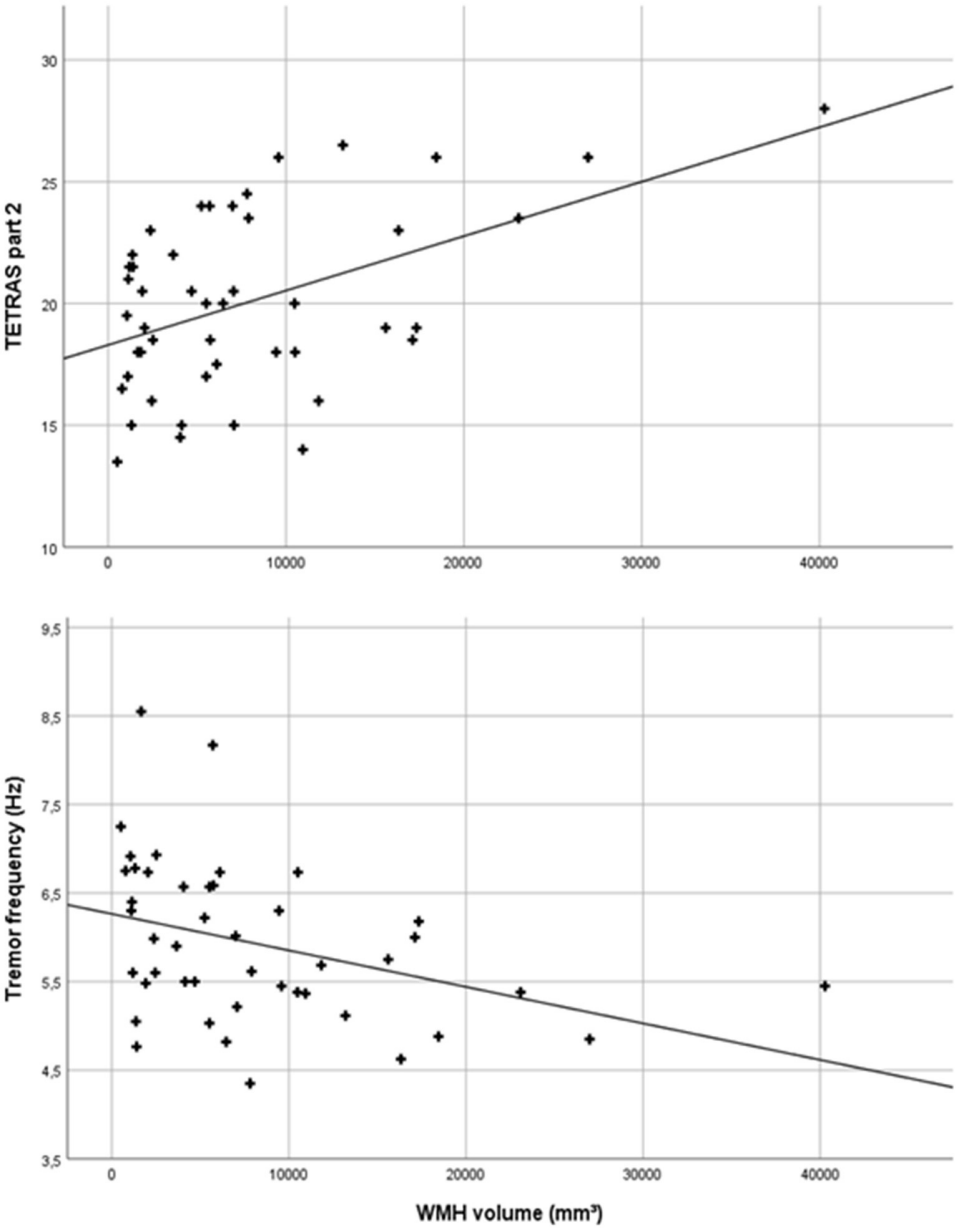
Scatter plot of total white matter hyperintensity (WMH) volumes and Tremor Research Group Essential Tremor Rating Assessment Scale (TETRAS) part 2 scores (*r* = 0.482, *p* = 0.001) and mean accelerometric tremor frequencies (*r* = −0.372, *p* = 0.012) of essential tremor (ET) patients.

The total WMH volume was significantly positively correlated with tremor severity on the TETRAS scale (TETRAS part 2: *r* = 0.482, *p* = 0.001; Figure 3), and there was a significantly negative correlation with the mean accelerometric tremor frequency (*r* = −0.372, *p* = 0.012; Figure 3), but there was no significant correlation between WMH volume and mean logarithmic total power.

A multiple linear regression model was compiled to predict tremor severity (TETRAS part 2) based on disease duration, age, and age-adjusted total WMH load. The adjusted *R*^2^ for the entire model was 0.279 [*F*_(3, 44)_ = 5.685, *p* = 0.002; Table 2]. Only the WMH volume significantly predicted tremor severity (β = 0.517, *p* < 0.001), while age and disease duration were not significant (each p > 0.05), meaning that higher WMH load was associated with more severe tremor. The same regression model with the mean tremor frequency as dependent variable showed an adjusted *R*^2^ for the whole model of 0.113 [*F*_(3, 44)_ = 2.873, *p* = 0.048; Table 2], and again, only the WMH volume significantly predicted tremor frequency (ß = 0.341, *p* = 0.02), while age and disease duration were not significant (each p > 0.05).

**Table 2:** Assumption-free ROI-based analysis to identify strategic white matter tracts that are associated with tremor severity (TETRAS part 2) and accelerometric frequency based on the JHU labels atlas.

		**TETRAS part 2**		**Frequency**	
**Regression model**	**Independent variables**	***R*^2^**	***p*-value *R*^2^**	***R*^2^**	***p*-value *R*^2^**
1	Age, disease duration	0.089	0.265	0.067	0.422
2	Model 1 + total WMH volume	**0.279**	**0.002**	**0.113**	**0.048**
3.1	Middle cerebellar peduncle	0.013	0.485	0.000	0.939
3.2	Pontine crossing tract	0.001	0.814	0.001	0.819
3.3	Genu corpus callosum	0.000	0.928	0.004	0.724
3.4	Body corpus callosum	0.100	0.047	0.045	0.201
3.5	Splenium corpus callosum	0.170	0.008	0.035	0.262
3.6	Fornix	0.004	0.712	0.006	0.644
3.7	Lemniscus medialis	0.025	0.329	0.000	0.984
3.8	Inferior cerebellar peduncle	0.008	0.590	0.000	0.925
3.9	Superior cerebellar peduncle	0.091	0.059	0.005	0.667
3.10	Cerebral peduncle	0.020	0.381	0.015	0.463
3.11	Anterior limb of CI	0.052	0.159	0.031	0.294
3.12	Posterior limb of CI	0.011	0.521	0.015	0.462
3.13	Retrolenticular part CI	0.047	0.178	0.02	0.325
3.14	Anterior corona radiata	0.078	0.081	0.042	0.216
3.15	Superior corona radiata	0.063	0.119	0.050	0.178
3.16	Posterior corona radiata	0.108	0.038	0.108	0.044
3.17	Posterior thalamic radiation	0.004	0.715	0.015	0.457
3.18	Sagittal Stratum	0.048	0.176	0.076	0.095
3.19	Cingulum	0.095	0.054	0.065	0.123
3.20	Cingulum hippocampal part	0.040	0.214	0.011	0.538
3.21	External Capsule	0.008	0.589	0.001	0.871
3.22	Fornix stria terminalis	0.011	0.516	0.002	0.805
3.23	Tapetum	0.000	0.906	0.001	0.850
3.24	Superior fronto-occipital fasciculus	0.224	0.002	0.093	0.063
3.25	Corticospinal tract	0.010	0.889	0.014	0.480
3.26	Superior longitudinal fasciculus	0.007	0.670	0.000	0.960
3.27	Uncinate fasciculus	0.007	0.607	0.008	0.596

*JHU, Johns Hopkins University; ROI, region of interest; TETRAS, Tremor Research Group Essential Tremor Rating Assessment Scale; WMH, white matter hyperintensity; CI, capsula interna; Significant values are marked in bold*.

The results of the assumption-free ROI-based analysis to identify strategic white matter tracts associated with tremor severity are shown in Table 2 and Supplementary Table 1. Age and disease duration do not significantly predict tremor severity or frequency (Regression-model 1, Table 2). After entering the age-adjusted WMH volume, the model becomes significant (Regression-model 2). Separate regression models for 27 white matter tracts after correction for total WMH volume were performed (significance level for model 3.1–3.27, *p* < 0.0019). After multiple comparison correction, none of the ROI significantly predicted the tremor measures.

## Discussion

In this study, the total and tract-specific amounts of WMHs were studied in a large sample of elderly ET patients between 60 and 84 years of age and age-matched healthy controls. While no group difference between ET patients and controls for total or ROI-based WMH load was found, there was a direct correlation between WMH load and tremor severity. Moreover, a multiple linear regression model showed that only the WMH load significantly predicted tremor severity and frequency, while age and disease duration had no significant effect.

Our study for the first time found evidence for a direct association between WMH and tremor severity. Within our sample of elderly ET patients, age *per se* was not directly associated with tremor severity, but the WMH load was. Our data suggest that WMHs contribute to the variability of tremor severity in the elderly and that WMH could be one factor among others mediating the relationship between biological aging and worsening of ET. Interestingly, we found no group differences between early-onset and late-onset ET patients and healthy controls regarding total or ROI-specific WMH volumes after age correction. This would imply that differences of WMH are not a relevant factor for the development of ET, no matter if earlier or later tremor onset. However, in patients with ET, the presence of WMH may impact the tremor severity.

DTI studies have shown widespread white matter microstructural alterations localized to cerebellar peduncles and pontine tracts as well as corticospinal tract and thalamo-cortical visual pathways in ET patients compared with healthy controls or patients with Parkinson's disease tremor (37–40). These studies differ methodologically from our study, since they examined DTI measures, while we quantified FLAIR hyperintensities and located them to certain fiber tracts. It is unclear if lesions localized on these specific tracts that were found abnormal in ET patients compared with healthy controls or patients with PD tremor are responsible for the association with tremor severity. Therefore, we chose an assumption-free ROI-based analysis using the JHU tracts and JHU labels atlases, but we were not able to localize strategic white matter tracts after adjusting for the total WMH volume. Therefore, pathophysiological conclusions from our findings remain limited.

To interpret these results appropriately, the relation of the physiological features of tremor frequency and amplitude and the morphological features of WMHs and brain lesions have to be considered: For ET, several studies have shown that aging is associated with an increase of tremor amplitude and a decrease of tremor frequency, independent of disease duration (8–10), but the underlying mechanism for this is not clear. Elble (8, 9) proposed that biological aging influences the symptom progression of ET by causing a gradual reduction in tremor frequency, which secondarily increases the amplitude of tremor. Tremor amplitude, frequency, and motor unit entrainment are logarithmically related to each other, and frequency and motor unit entrainment make comparable and independent contributions to tremor amplitude (41).

It is well-documented that strategic lesions can produce different kinds of tremors (42). Lesions within the cerebellum and particularly the upper cerebellar peduncle may cause intention-tremor syndromes (43–45). Midbrain lesions near the rubral and subthalamic nucleus have long been described to produce specific rest and intention tremors as documented by Benedikt (46) and Holmes (47). Thalamic lesions may produce similar tremors (48), although they are mostly accompanied by dystonic or other hyperkinetic symptoms. The tremor-producing effect of lesions is usually explained by destruction of motor centers being responsible for damping oscillations like the cerebellum or the pallidum (3, 49). On the other hand, brain lesions can abruptly stop tremors. Lesions, particularly ischemic strokes in specific areas like the ventrolateral thalamus, can alleviate preexisting ET well-known from neurosurgical interventions (50). More generally, if such lesions occur along the pathway of the tremor network of ET (16, 51), the cerebello-thalamo-cortical projection or within the cortico-spinal tract, a preexisting tremor can be extinguished (52). The tremor reduction following lesions is assumed to result from destruction of pathways that mediate tremor excitations (53).

The mechanism underlying the tremor-modulating effect of WMH is likely to differ from these better established mechanisms and is mostly speculative: WMHs correspond pathologically to areas of demyelination and gliosis (18). Axonal demyelination causes a decrease in nerve conduction velocity or even a total conduction block of action potentials. Partial conduction blocks are typically frequency related. While high-frequency impulses are not transmitted, low-frequency impulses may still reliably pass through (54). Essential tremor in elderly patients is characterized by low-frequency tremor (8). This low frequency leads to an elevated tremor amplitude, which causes the functional impairment. Increased WMH load reflects locally distributed demyelination and gliosis (18), and it is conceivable that this leads to an abnormal processing of high-frequency impulses. The result would be a decrease of tremor frequency and increase of tremor amplitude and functional impairment. Thus, WMHs might directly modulate centrally generated tremor frequencies. Of course, this explanation remains speculative and requires further investigations.

The limitations of our study are that no longitudinal data have been recorded to support the potentially causative relation between WMH and tremor severity. Apart from disease duration, age, and WMH, several other factors that have not been identified yet might potentially affect tremor severity in the elderly and these might not be captured by our regression models. Disease duration was calculated retrospectively from the patient-reported age at symptom onset, but this information typically does not mirror the “real” disease onset because many patients do not recognize their condition when it is mild (55). We included ET patients with and without additional neurological signs of uncertain significance (ET plus), and subgroups of ET patients were carefully compared with each other regarding total or ROI-specific WMH volumes. But since we found no group differences, all ET patients were pooled for further analyses to increase statistical power. So far, it is not clear if certain ET plus subtypes differ from ET patients regarding total or regional WMH load, and our study is not powered to finally answer this question. Therefore, future studies should explore brain structural differences in larger subgroups of ET patients. Our patient and control groups differed in their MoCA scores, even after correction for age and school education. These findings are in line with the literature, since several clinical and epidemiological studies have shown poorer cognitive performance in ET patients compared with healthy controls and additionally, an increased risk to develop dementia (12, 56–58). A concerning limitation is that we cannot reproduce the previous finding of a higher WMH load in patients compared to controls. (22). A possible explanation is the smaller sample size in our study. *Vice versa*, the previous study (22) did not report the tremor amplitude/WMH relation, which was not part of their protocol and the diagnosis of ET was established on handwriting samples. Apart from the clinical tremor assessment (TETRAS), we performed a polygraphic tremor analysis, since we aimed for an electrophysiologic outcome parameter as well. Interestingly, tremor frequency seemed to more adequately reflect tremor severity, since TETRAS scores and frequency measures were significantly negatively correlated in our patients. Therefore, we chose to consider tremor frequency instead of accelerometric total power as an electrophysiological parameter additionally to the TETRAS score. Finally, we were not able to localize the clinical effect of WMH on tremor characteristics to a strategic white matter tract of the JHU atlas. Most possibly this is due to the small sample size. Lesion–symptom studies on WMHs typically require hundreds of individuals (59).

We conclude that total or ROI-specific WMHs are not differing between ET patients and controls, but we have a relatively robust relation between tremor severity and the WMH load. Our data provide the first evidence for a worsening of ET in the presence of WMHs in the elderly. The WMH load might be one factor among others mediating the tremor severity in (disposed) elderly ET patients, and this could at least partly explain the well-established relation between aging and increase of tremor severity. However, these findings need to be confirmed in larger studies.

## Data Availability Statement

The raw data supporting the conclusions of this article will be made available by the authors, without undue reservation.

## Ethics Statement

The studies involving human participants were reviewed and approved by Ethics Committee of Christian Albrechts University Kiel. The patients/participants provided their written informed consent to participate in this study.

## Author Contributions

GD, DB, KZ, and JSB designed the study. JSB, JB, UJ-K, and SW organized and executed the study. JSB, OG, and IT performed analysis and interpreted the results. JSB and JB wrote the first draft of the manuscript. All authors contributed to the article and approved the submitted version.

## Conflict of Interest

KZ has received research support from an intramural grant from the Christian-Albrechts University of Kiel, from the Benign Essential Blepharospasm Foundation, and with an unrestricted grant from Ipsen. She reports speaker's honoraria from Bayer Vital GmbH, AbbVie Allergan, and Merz outside the submitted work. She has served as a consultant and received fees from Merz, Ipsen, and the German Federal Institute for Drugs and Medical Devices (BfArM). DB has served on scientific advisory boards for UCB Pharma GmbH and Lundbeck; has received speaker honoraria from UCB Pharma GmbH, Lundbeck, BIAL, Bayer, AbbVie, Biogen, and Zambon; and has received grants from Michael J. Fox Foundation, Janssen Pharmaceutica N.V., German Parkinson's Disease Association, BMWi, BMBF, ParkinsonFonds Deutschland gGmbH, UCB Pharma GmbH, TEVA Pharma GmbH, EU, Novartis Pharma GmbH, Lundbeck and Damp foundation. GD received fees for lecturing from Boston Scientific and consulting fees from Boston Scientific, Cavion, Functional modulation. He receives funding for his research to his institution from the German Research Council, SFB 1261, B5, and Medtronic. The remaining authors declare that the research was conducted in the absence of any commercial or financial relationships that could be construed as a potential conflict of interest.
