# Supplementary material for: White Matter Hyperintensities Are Associated With Severity of Essential Tremor in the Elderly
Source: Front Neurol. 2021 Jun 28;12:694286. doi: 10.3389/fneur.2021.694286 (PMC8273287; doi:10.3389/fneur.2021.694286)
Supplement: Supplementary file 1 [file Table_1.DOCX]

**Supplementary Table 1.** Assumption-free ROI-based analysis to identify strategic white matter tracts that are associated with tremor severity (TETRAS part 2) and accelerometric frequency based on the JHU tracts atlas.

|  |  | **TETRAS part 2** | | **Frequency** | |
| --- | --- | --- | --- | --- | --- |
| **Regression- model** | **Independent variables** | **R²** | **p-value R²** | **R²** | **p-value R²** |
| **1** | **Age, disease duration** | 0.089 | 0.265 | 0.067 | 0.422 |
| **2** | **Model 1 + total WMH volume** | **0.279** | **0.002** | **0.113** | **0.048** |
| **3a** | **WMH volume anterior thalamic radiatio** | 0.150 | 0.012 | 0.103 | 0.046 |
| **3b** | **WMH volume corticospinal tract** | 0.005 | 0.672 | 0.001 | 0.874 |
| **3c** | **WMH volume cingulum of cingulate gyrus** | 0.005 | 0.663 | 0.105 | 0.044 |
| **3d** | **WMH volume cingulum of hippocampus** | 0.050 | 0.160 | 0.054 | 0.156 |
| **3e** | **WMH volume inferior fronto-occipital fasciculus** | 0.033 | 0.253 | 0.000 | 0.928 |
| **3f** | **WMH volume inferior longitudinal fasciculus** | 0.131 | 0.020 | 0.016 | 0.443 |
| **3g** | **WMH volume superior longitudinal fasciculus** | 0.096 | 0.049 | 0.051 | 0.168 |
| **3h** | **WMH volume uncinate fasciculus** | 0.103 | 0.041 | 0.042 | 0.209 |
| **3i** | **WMH volume temporal part of superior longitudinal fasciculus** | 0.086 | 0.063 | 0.086 | 0.070 |
| **3j** | **WMH volume forceps major** | 0.015 | 0.449 | 0.002 | 0.798 |
| **3k** | **WMH volume forceps minor** | 0.037 | 0.225 | 0.002 | 0.797 |
| Age and disease duration do not significantly predict tremor severity or frequency (Regression-model 1). After entering WMH volume, the model becomes significant (Regression-model 2). Regression-models 3a-k: Separate regression models for 11 white matter tracts after correction for total WMH volume (significance level for model 3a-k: p<0.0045) | | | | | |
